# Healthy lifestyles are associated with better vitamin D status in community‐dwelling older men: The Health In Men Study (HIMS)

**DOI:** 10.1111/cen.14926

**Published:** 2023-05-10

**Authors:** Xiaoying Liu, Kaye E. Brock, Tara C. Brennan‐Speranza, Leon Flicker, Jonathan Golledge, Graeme J. Hankey, Christian M. Girgis, Bu B. Yeap

**Affiliations:** ^1^ Faculty of Medicine and Health The University of Sydney Sydney Australia; ^2^ Medical School University of Western Australia Perth Australia; ^3^ Western Australian Centre for Health & Ageing University of Western Australia Perth Australia; ^4^ Queensland Research Centre for Peripheral Vascular Disease James Cook University Townsville Australia; ^5^ Department of Vascular and Endovascular Surgery Townsville University Hospital Townsville Australia; ^6^ Perron Institute for Neurological and Translational Science Perth Australia; ^7^ Department of Diabetes and Endocrinology Westmead Hospital Sydney Australia; ^8^ Department of Endocrinology and Diabetes Fiona Stanley Hospital Perth Australia

**Keywords:** aged, exercise, healthy aging, healthy lifestyle, men, vitamin D

## Abstract

**Objective:**

Older people are more prone to vitamin D deficiency than younger populations. Individual lifestyle factors have been associated with vitamin D status. We examined the influence of a combination of lifestyle factors on vitamin D status in older men.

**Participants and Measurements:**

In a population‐based cohort study of older men (age ≥65 years), a lifestyle score was calculated from eight prudent health‐related behaviours (smoking, exercise, alcohol, fish and meat consumption, adding salt, milk choices and obesity) collected via questionnaire at baseline. Blood samples were collected 5 years afterwards to measure plasma 25‐hydroxyvitamin D (25OHD) levels. Associations between lifestyles and the likelihood of having plasma 25OHD levels of ≥75 versus <75 nmol/L and ≥50 versus <50 nmol/L were tested using logistic regression models.

**Results:**

Of the 2717 men analysed, mean plasma 25OHD was 69.0 ± 23.5 nmol/L, with 20.7% having plasma 25OHD <50 nmol/L. Men engaging in ≥4 healthy lifestyle behaviours had 20% higher odds of plasma 25OHD ≥75 nmol/L (adjusted OR = 1.20, 95% CI: 1.01−1.45) compared to those with <4 healthy behaviours. No association was found for 25OHD ≥50 nmol/L. Higher physical activity was the only individual component significantly associated with vitamin D sufficiency (highest vs. lowest quintiles of physical activity, adjusted OR = 2.01, 95% CI: 1.47−2.74 for 25OHD ≥50 nmol/L, adjusted OR = 2.35, 95% CI: 1.81−3.06 for 25OHD ≥75 nmol/L).

**Conclusion:**

Multiple healthy lifestyle behaviours are associated with better vitamin D status in older men. Further work is needed to determine the effects of promoting healthy lifestyle behaviours, including physical activity, on vitamin D sufficiency.

## 1 INTRODUCTION

Bioactive vitamin D is a hormone that stimulates the gut absorption of calcium and phosphate and is essential for bone and muscle health.^1^ There are two major forms of vitamin D. Vitamin D_3_ is mainly synthesized in the skin after the exposure to sunlight, whereby 7‐dehydrocholesterol in the skin is converted to pre‐vitamin D_3_ during ultraviolet B radiation and is then immediately converted to vitamin D_3_.^1^ Latitude, season, skin pigmentation, use of sunscreen and clothing cover, and outdoor activities that affect sunlight exposure can influence dermal synthesis of vitamin D_3_.^1^ Small amounts of vitamin D are found in food such as oily fish, meat, eggs, mushrooms (a source of the other form, vitamin D_2_) and vitamin D fortified food.^2^ The blood concentration of the intermediate metabolite, 25‐hydroxyvitamin D (25OHD), is used to determine vitamin D status. Severe deficiency of vitamin D, characterized by 25OHD levels below 25 nmol/L, can have detrimental effects on health, including impaired bone mineralization.^3^

Endogenous synthesis of vitamin D declines with aging partly due to a reduction in skin 7‐dehydrocholesterol, with effects on calcium absorption further exacerbated by reduced renal production of the bioactive form, 1,25‐dihydroxyvitamin D (1,25(OH)_2_D).^4^ Furthermore, decreased endogenous synthesis of vitamin D is accentuated in older people with sedentary behaviours and limited sunlight exposure, particularly those who are institutionalized, or house bound.^1^ The decrease in vitamin D production and calcium absorption together lead to increased risk of negative calcium balance and bone loss in older‐aged populations.^5^ In recent years, vitamin D deficiency and insufficiency has been recognized as an important public health issue worldwide.^3^ Australia is known for its abundance of sunshine, with most of the population obtaining vitamin D through cutaneous synthesis during sun exposure. However, the national survey indicated a lower prevalence of vitamin D deficiency (25OHD <50 nmol/L) in older age groups compared to younger groups.^6^ This difference can be partially attributed to the higher likelihood of older individuals taking vitamin D supplements.^6^ However, a significant number of falls‐ and hip fracture‐related hospitalizations in the elderly Australian population remain attributable to vitamin D deficiency.^7^

In Australia, serum 25OHD level ≥50 nmol/L by the end of winter is considered adequate,^8^ consistent with recommendations from the Institute of Medicine in United States of America.^9^ In contrast, the UK Scientific Advisory Committee on Nutrition recommends a lower limit of vitamin D adequacy, described as a ‘population protective’ concentration with a serum 25OHD ≥25 nmol/L that should be achieved by 97.5% of the population throughout the year.^10^ Other health institutes and organizations advocate a higher threshold of 75 nmol/L for the general population.^11^ However, there is limited evidence to support higher vitamin D targets.^8^, ^12^ The European Society for Clinical and Economic Aspects of Osteoporosis and Osteoarthritis suggested the oldest old (aged >80 years) and frail older people with increased falls and fractures risk should have serum 25OHD levels of at least 75 nmol/L.^13^

Healthy lifestyle behaviours, such as regular exercise, and oily fish consumption, are beneficial for bone and muscle health; and these lifestyle factors have been associated with increased blood levels of 25OHD.^1^ However, these factors have been studied individually rather than as part of a multi‐lifestyle behavioural pattern. Thus, the aim of this study was to investigate the association between healthy lifestyles (a combination of behaviours) and circulating 25OHD levels in a large population of older, community‐dwelling men.

## 2 METHODS

### 2.1 Study population

The study cohort comprised men who participated in the Health In Men Study (HIMS), a population‐based randomized trial of screening for abdominal aortic aneurysms in Perth, Western Australia.^14^ Men aged 65 years and older were randomly selected from the electoral roll and recruited into the trial in 1996−1999 (Wave 1). There were 12,203 men who completed the baseline survey that covered aspects of medical history and lifestyle relevant to CVD. In the follow‐up survey conducted in 2001−2004 (Wave 2), 4248 men were reassessed and had an early morning blood sample collected, in which plasma 25OHD was measured. Information on vitamin D supplementation was also collected at Wave 2. The human research ethics committee of the University of Western Australia approved the study protocol, and all study participants gave their written informed consent. This study is a post hoc analysis of a lifestyle score assessed at Wave 1 and plasma 25OHD levels measured during Wave 2.

### 2.2 Lifestyle behaviour and prudent lifestyle score

Lifestyle information was collected via a questionnaire in Wave 1 (1996−1999). Physical activity levels were assessed by asking study participants how much time they spent performing non‐vigorous (moderate) and vigorous exercise separately. A metabolic equivalent (MET) value of three was assigned to non‐vigorous activity and a MET value of five was assigned to vigorous activity. As such, total MET hours per week were then calculated as the sum of the hours of non‐vigorous activity multiplied by three and the hours of vigorous activity multiplied by five.^15^ Alcohol consumption was assessed by asking the number of daily alcohol drinks in a week. Weekly fish and meat consumption were assessed by asking the participants to select from five options: (1) six or more times per week; (2) three to five times per week; (3) one or two times per week; (4) less than once per week; (5) never. Salt intake was similarly assessed by asking participants to select from three options: (1) rarely or never; (2) sometimes; (3) almost always or always. Type of milk was assessed by selecting from six options: (1) condensed; (2) full cream; (3) sometimes full cream and skim/reduced fat; (4) reduced fat; (5) skim or none; (6) other.

Lifestyle scores were then calculated from eight behaviours, with a score allocated for each ‘healthy’ behaviour: (1) having never smoked or having stopped smoking more than a year ago; (2) doing minimum of 3 h a week of at least non‐vigorous (moderate) physical activity; (3) having less than 6 alcoholic drinks/day and no more than 28/week; (4) eating fish at least three times weekly; (5) eating meat less than six times weekly; (6) never or rarely adding salt to food; (7) always using reduced fat or skim milk; (8) having a measured BMI of <25 kg/m^2^. The resulting scores ranged from 0 to 8, with a higher score representing a healthier lifestyle. The prudent lifestyle score has been validated in previous studies that have shown these lifestyle scores predict survival and vascular diseases in the same population.^16^, ^17^ In the present study, scores of 7 and 8 were combined to provide adequate group sizes for each score category.

### 2.3 Laboratory assays

Blood samples were collected between 8 am and 10:30 am. Information on fasting status was also collected. Aliquots of serum and plasma were prepared immediately after phlebotomy and stored at −80°C until assayed. Plasma 25OHD concentrations were measured with an automated ‘DiaSorin Liaison 25(OH)D total’ chemiluminescent immunoassay between 2011 and 2012. The interassay coefficient of variation of the assay was 13.2% at 37.9 nmol/L and 11.3% at 131 nmol/L. The date of the blood sample collection was recorded and categorized into spring (September to November), summer (December to February), autumn (March to May) and winter (June to August). Plasma 25OHD levels were then categorized using two cut‐offs: 50 and 75 nmol/L. Plasma 25OHD ≥50 nmol/L at the end of winter is the current definition of vitamin D adequacy in Australia.^8^ Considering the older age of our study subjects with substantially higher requirements for vitamin D and increased risk of fracture and osteoporosis, plasma 25OHD ≥75 nmol was also investigated in the present analysis.

### 2.4 Statistical analysis

Descriptive characteristics of the study population were expressed as mean ± SD for continuous variables and *n* (%) for categorical variables, stratified by plasma 25OHD levels <50, 50−74.9 and ≥75 nmol/L. One‐way analysis of variance was used to compare continuous variables (age and BMI) between stratification of plasma 25OHD levels <50, 50−74.9 and ≥75 nmol/L, and the Pearson's *χ* ^2^ statistic was applied for comparison of categorical variables. Linear regression analyses were performed to determine the relationship between prudent lifestyle score categories and continuous plasma 25OHD levels. Results were presented as β and corresponding 95% confidence interval (95% CI). Logistic regression analyses were applied to determine the relationship between prudent healthy lifestyle scores and odds of having plasma 25OHD levels greater than 50 and greater than 75 nmol/L. Results were presented as odds ratio (OR) and 95% CI. Adjustment was made for age, season of blood draw, time difference between baseline survey and blood collection, metabolic syndrome (MetS) and vitamin D supplementation status. Linear and logistic regression analyses were then repeated to determine the relationship between individual lifestyle factors and vitamin D status. All statistical analysis was performed with the statistical software R (R Foundation for Statistical Computing; Version 4.0.3). Statistical significance was defined as *p* value below .05.

## 3 RESULTS

### 3.1 Population characteristics

Table 1 presents the demographic, anthropometric, clinical and lifestyle characteristics of the study participants. Of the 4248 men who participated and donated a blood sample at Wave 2, excluding men with missing data and those who were not fasted at the time of blood collection, a total of 2717 had complete data for fasted plasma 25OHD levels and lifestyle behaviours and were included in the current analysis. Mean (±SD) age of these men was 76.5 ± 3.5 years. The mean BMI was 26.7 ± 3.2 kg/m^2^ at Wave 1 and only 32% of men had a BMI < 25 kg/m^2^.

**Table 1:** Demographic, anthropometric, clinical and lifestyle characteristics of the study population, stratified by vitamin D status.

	Total	Plasma 25OHD <50 nmol/L	Plasma 25OHD 50−74.9 nmol/L	Plasma 25OHD ≥75 nmol/L	*p* Values
*N*	2717	562 (20.7%)	1163 (42.8%)	992 (36.5%)	
Plasma 25OHD levels (nmol/L)	69.0 ± 23.5	39.3 ± 8.5	63.2 ± 7.1	92.6 ± 17.7	
Age (years)	76.5 ± 3.5	77.0 ± 3.7	76.4 ± 3.5	76.3 ± 3.5	<.001
Vitamin D supplementation					
Yes	22 (0.8%)	7 (1.2%)	8 (0.7%)	7 (0.7%)	.43
No	2695 (99.2%)	555 (98.8%)	1155 (99.3%)	985 (99.3%)	
Education					.78
Completed high school or higher education	1384 (51%)	287 (51.1%)	584 (50.2%)	513 (51.7%)	
Did not complete high school or higher education	1333 (49%)	275 (48.9%)	579 (49.8%)	479 (48.3%)	
BMI at baseline (kg/m^2^)	26.7 ± 3.2	27.0 ± 3.7	26.8 ± 3.2	26.3 ± 3.0	<.001
Season of blood collection					<.001
Spring	585 (21.5%)	162 (27.7%)	263 (45.0%)	160 (27.4%)	
Summer	377 (13.9%)	57 (15.1%)	176 (46.7%)	144 (38.2%)	
Autumn	938 (34.5%)	121 (12.9%)	354 (37.7%)	463 (49.4%)	
Winter	817 (30.1%)	222 (27.2%)	370 (45.3%)	225 (27.5%)	
Medical history					
Metabolic syndrome	819 (30.1%)	218 (38.8%)	372 (32.0%)	229 (23.1%)	<.001
Individual lifestyle behaviour					
Never smoked or stopped >1 year	1823 (67.1%)	397 (70.6%)	764 (65.7%)	662 (66.7%)	.12
Exercise ≥3 h/week	1763 (64.9%)	336 (59.8%)	714 (61.4%)	713 (71.9%)	<.001
<6 alcohol drinks/day and ≤28 drinks/week	2485 (91.5%)	517 (92.0%)	1054 (90.6%)	914 (92.1%)	.40
Eat fish ≥3 times/week	250 (9.2%)	65 (11.6%)	92 (7.9%)	93 (9.4%)	.05
Eat meat <6 times/week	1873 (68.9%)	383 (68.1%)	802 (69.0%)	688 (69.4%)	.89
Never or rarely add salt to food	1091 (40.2%)	221 (39.3%)	480 (41.3%)	390 (39.3%)	.59
BMI <25 kg/m^2^	867 (31.9%)	170 (30.2%)	353 (30.4%)	344 (34.7%)	.06
Always use reduced fat/skim milk	1458 (53.7%)	287 (51.1%)	620 (53.3%)	551 (55.5%)	.22

*Note*: Data was expressed as mean ± SD for continues variables and number (percentage) for categorical variables.

In terms of healthy lifestyle behaviours, there were 1823 (67.1%) men who had quit (*n* = 1607) or never smoked (*n* = 216). Sixty‐five percent of study participants achieved moderate physical activity of at least 3 h per week. Most men consumed less than 6 alcoholic drinks per day or no more than 28 drinks per week (91.5%) and ate meat less than 6 times per week (68.9%). Less than half of the men reported salt intake limited to never or rarely adding salt to food (40.2%) and half always used reduced fat milk (53.7%). Only 9.2% men reported fish consumption more than three times per week.

### 3.2 Vitamin D status

Overall, the average plasma 25OHD level was 69.0 ± 23.5 nmol/L with lower levels during Winter and Spring seasons (spring: 63.5 ± 20.8 nmol/L; summer: 71.7 ± 21.6 nmol/L; autumn 76.3 ± 25.4 nmol/L; winter: 63.3 ± 21.3 nmol/L; *p* < .05). Over one‐fifth of participants had plasma 25OHD levels below 50 nmol/L; this proportion was higher in Winter (27.2%) and Spring (27.7%) seasons compared to the Summer (15.1%) and Autumn (12.9%). There were 1163 (42.8%) men with plasma 25OHD levels between 50 and 74.9 nmol/L and 992 (36.5%) with plasma 25OHD levels greater than 75 nmol/L. Men with vitamin D <50 nmol/L were older, had higher BMI, were more likely to have MetS or type 2 diabetes, and were less physically active (Table 1).

### 3.3 Association between lifestyles scores and subsequent vitamin D status

Table 2 presents the linear and logistic regression analysis between lifestyle score and plasma 25OHD levels and odds of vitamin D sufficiency (≥50 and ≥75 nmol/L) for incremental increases in lifestyle score and when categorized as scores <4 and ≥4. Mean plasma 25OHD levels increased with higher lifestyle scores. In the univariate analysis, men who engaged in six or more healthy lifestyle behaviours had higher plasma 25OHD compared to men with a score of one. This association was no longer significant in the multivariable model. When lifestyle scores were grouped into two categories, men who engaged in four or more healthy lifestyle behaviours had a significantly higher plasma 25OHD compared to men with lifestyle score <4. After adjustment for age, season of blood draw, time difference between baseline survey and blood collection, MetS and vitamin D supplementation, the association was no longer significant.

**Table 2:** Association between prudent lifestyle score and vitamin D adequacy.

	Mean plasma 25OHD levels	Continuous plasma 25OHD levels^a^		Plasma 25OHD ≥50 nmol/L^b^		Plasma 25OHD ≥75 nmol/L^b^	
		Univariate	Multivariable^c^	Univariate	Multivariable^c^	Univariate	Multivariable^c^
Lifestyle score							
≤1	64.4 ± 21.9	Reference	Reference	Reference	Reference	Reference	Reference
2	68.1 ± 24.6	3.7 (−3.7 to 11.1)	3.0 (−4.9 to 10.1)	1.11 (0.52−2.34)	1.02 (0.47−2.20)	0.95 (0.48−1.88)	0.91 (0.45−1.83)
3	67.6 ± 23.5	3.3 (−3.8 to 10.3)	3.1 (−3.7 to 9.8)	1.11 (0.55−2.25)	1.07 (0.51−2.21)	1.08 (0.57−2.04)	1.08 (0.56−2.09)
4	68.3 ± 22.4	3.9 (−3.0 to 10.8)	3.2 (−3.4 to 9.8)	1.18 (0.59−2.38)	1.09 (0.53−2.24)	1.19 (0.63−2.24)	1.16 (0.60−2.23)
5	69.1 ± 22.9	4.7 (−2.2 to 11.6)	3.7 (−2.9 to 10.4)	1.19 (0.5−2.39)	1.07 (0.52−2.21)	1.32 (0.70−2.48)	1.26 (0.66−2.43)
6	71.9 ± 24.9^d^	7.5 (0.4−14.6)^d^	6.1 (−0.7 to 12.9)	1.25 (0.61−2.57)	1.12 (0.53−2.36)	1.40 (0.73−2.68)	1.29 (0.66−2.53)
≥7	72.8 ± 27.0^d^	8.5 (0.5−16.4)^d^	6.1 (−1.5 to 13.7)	1.26 (0.56−2.85)	1.02 (0.44−2.37)	1.80 (0.88−3.67)	1.58 (0.75−3.30)
Grouped							
<4	67.6 ± 23.7	Reference	Reference	Reference	Reference	Reference	Reference
≥4	69.6 ± 23.4^d^	2.0 (0.1−3.9)^d^	1.3 (−0.6 to 3.1)	1.09 (0.89−1.34)	1.03 (0.84−1.28)	1.26 (1.06−1.51)^d^	1.20 (1.01−1.45)^d^

Abbreviation: MetS, metabolic syndrome.

^a^ Results presented as β coefficient.

^b^ Results presented as odds ratio (OR).

^c^ Adjusted for age, season of blood draw, time between lifestyle survey and blood draw, MetS and vitamin D supplementation.

^d^ *p* < .05 test by linear regression or logistic regression.

In both univariate and multivariable analysis, higher categories of lifestyle score did not predict thresholds of plasma 25OHD ≥50 or ≥75 nmol/L. When lifestyle scores were grouped into two categories, engagement in four or more healthy lifestyle behaviours was not associated with odds of having subsequent total plasma 25OHD ≥50 nmol/L compared to men with lifestyle score <4. When the threshold of vitamin D was increased to 75 nmol/L, older men with a healthy lifestyle score of at least four had higher odds of plasma 25OHD ≥75 nmol/L (adjusted OR = 1.20, 95% CI: 1.01−1.45).

### 3.4 Individual lifestyles behaviours and subsequent vitamin D status

Smoking and dietary components of healthy lifestyles, including non‐excessive alcohol consumption, eating more fish, less meat and lower salt intake and use of reduced fat/skim milk, as well as obesity status, were not associated with plasma 25OHD levels, nor odds of plasma 25OHD being ≥50 or ≥75 nmol/L (Table 3).

**Table 3:** Association between individual lifestyle behaviour and plasma 25OHD levels and odds of vitamin D deficiency (plasma 25OHD ≥50 or ≥75 nmol/L).

	Plasma 25OHD levels		Plasma 25OHD ≥50 nmol/L		Plasma 25OHD ≥75 nmol/L	
	Mean ± SD	Multivariable^a^^,^ b	Univariate	Multivariable^a^^,^ c	Univariate	Multivariable^a^^,^ c
Smoking						
Current smoker	69.5 ± 22.8	Reference	Reference	Reference	Reference	Reference
Never smoked/quit	68.7 ± 23.8	0.1 (−1.8 to 1.9)	0.81 (0.66−1.00)*	0.85 (0.69−1.05)	0.97 (0.83−1.15)	1.03 (0.87−1.22)
Physical activity (total MET h/week)						
Q1 (≤2.5)	65.3 ± 24.3	Reference	Reference	Reference	Reference	Reference
Q2 (2.6−11.5)	66.3 ± 21.0	1.7 (−1.0 to 4.4)	1.30 (0.98−1.71)	1.38 (1.04−1.84)*	1.12 (0.87−1.43)	1.18 (0.90−1.55)
Q3 (11.6−20.0)	69.2 ± 23.2*	4.1 (1.4−6.7)*	1.42 (1.07−1.88)*	1.50 (1.12−2.00)*	1.44 (1.12−1.84)*	1.51 (1.16−1.97)*
Q4 (20.1−31.5)	72.3 ± 24.3*	5.7 (3.0−8.4)*	1.60 (1.19−2.14)*	1.67 (1.23−2.26)*	1.80 (1.38−2.35)*	1.89 (1.45−2.47)*
Q5 (>31.5)	75.2 ± 24.9*	9.0 (6.3−11.7)*	1.99 (1.48−2.70)*	2.01 (1.47−2.74)*	2.40 (1.82−3.18)*	2.35 (1.81−3.06)*
Alcohol						
≥6/day or ≥28/week	67.8 ± 20.2	Reference	Reference	Reference	Reference	Reference
<6/day or <28/week	69.1 ± 23.8	1.5 (−1.5 to 4.5)	0.92 (0.65−1.29)	0.93 (0.66−1.33)	1.15 (0.86−1.53)	1.16 (0.86−1.56)
Fish						
Never	70.1 ± 38.3	Reference	Reference	Reference	Reference	Reference
<1/week	69.4 ± 23.6	−2.4 (−10.1 to 5.4)	2.28 (1.10−4.73)*	2.07 (0.97−4.40)	0.92 (0.45−1.88)	0.80 (0.38−1.67)
1−2 times/week	69.0 ± 22.9	−2.9 (−10.6 to 4.7)	2.16 (1.06−4.40)*	1.95 (0.93−4.08)	0.93 (0.46−1.86)	0.80 (0.39−1.65)
≥3 times per week	67.2 ± 24.7	−4.4 (−12.5 to 3.7)	1.55 (0.73−3.31)	1.37 (0.61−3.00)	0.96 (0.46−2.00)	0.82 (0.38−1.75)
Meat						
≥6 times/week	68.5 ± 23.4	Reference	Reference	Reference	Reference	Reference
<6 times/week	69.2 ± 23.5	0.7 (−1.2 to 2.5)	1.05 (0.86−1.28)	1.04 (0.85−1.28)	1.03 (0.87−1.22)	1.03 (0.86−1.22)
Add salt						
Sometimes or always	68.9 ± 23.6	Reference	Reference	Reference	Reference	Reference
Rarely or never	69.1 ± 23.4	−0.5 (−2.2 to 1.3)	1.04 (0.86−1.26)	1.01 (0.83−1.22)	0.95 (0.81−1.11)	0.90 (0.76−1.06)
Milk						
Condense/full cream	68.6 ± 24.4	Reference	Reference	Reference	Reference	Reference
Reduced fat/skim	69.3 ± 22.7	0.2 (−1.5 to 1.9)	1.14 (0.95−1.37)	1.10 (0.91−1.34)	1.13 (0.96−1.32)	1.10 (0.93−1.29)
BMI (kg/m^2^)						
≥25	67.8 ± 22.8	Reference	Reference	Reference	Reference	Reference
<25	71.5 ± 24.7*	1.9 (0.1−3.8)*	1.10 (0.90−1.35)	0.95 (0.76−1.18)	1.22 (1.03−1.44)*	1.06 (0.89−1.27)

Abbreviation: MetS, metabolic syndrome.

^a^ Adjusted for age, season of blood draw, time between lifestyle survey and blood draw, MetS and vitamin D supplementation.

^b^ Results presented as β coefficient.

^c^ Results presented as odds ratio (OR).

* *p* < .05.

Physical activity level was the only individual lifestyle behaviour that was associated with subsequent higher plasma 25OHD levels. When physical activity was expressed as total MET hours per week, higher total MET hours was associated with higher plasma 25OHD concentrations and increased odds of plasma 25OHD ≥50 or ≥75 nmol/L (Table 3; Q5 [>31.5 MET h/week] vs. Q1 [≤2.5 MET h/week], OR = 2.01, 95% CI: 1.47−2.74 for plasma 25OHD ≥50 nmol/L; OR = 2.35, 95% CI: 1.81−3.06 for plasma 25OHD ≥75 nmol/L).

## 4 DISCUSSION

In this population of community‐dwelling older men, engaging in a range of healthy lifestyle behaviours was associated with greater likelihood of having plasma 25OHD ≥75 nmol/L. Having a higher level of physical activity was the only individual component of the lifestyle score which was associated with higher plasma 25OHD levels in this cohort of older men, who were mostly vitamin D sufficient.

Men in our study had a mean plasma 25OHD of 69.0 ± 23.5 nmol/L with 21% of the participants having plasma 25OHD <50 nmol/L. The average 25OHD concentration in our population was higher than a similar population at Sydney, Australia, which reported mean serum 25OHD level at 55.9 nmol/L and 43% who had vitamin D <50 nmol/L.^18^ However, the prevalence of vitamin D deficiency in our population remained higher than the reported national prevalence among men aged ≥75 years old (13.2%) in the 2011−2013 Australian Health Survey.^6^ One possible reason could be the differences in assay methods for 25OHD. The current study used a Diasorin Liaison total chemiluminescent immunoassay, whereas the CHAMP study used a radioimmunoassay,^18^ and the Australian Health Survey a liquid chromatography tandem mass spectrometry (LC‐MS/MS) assay. The Liaison immunoassay has been reported to underestimate 25OHD values compared to the LC‐MS/MS assay,^19^ which may partly explain the higher prevalence of 25OHD <50 nmol/L in our study compared to the nationwide Australian Health Survey. In addition, our study site (Perth: 31°S) was located at a higher latitude and thus is exposed to stronger UV radiation than the CHAMP study site (Sydney: 33.9°S).^6^ Also, differences in ethnicity of the study cohorts may explain the variance in vitamin D between studies. Participants of the present study were mainly Caucasian, while the CHAMP study and the Australian Health Survey include subjects with a wider range of ethnicities.^6^, ^18^ People with fair‐skin have greater synthetic ability for vitamin D_3_ synthesis from sunlight exposure; whereas fair‐to‐dark skinned and dark‐skinned people are more likely to have suboptimal vitamin D status due to the limited ability of vitamin D synthesis.^1^

In our population‐based study in a sunny location, 21% of the participants in our study had plasma 25OHD <50 nmol/L, and this proportion was 27% in winter. This is a public health concern, given the higher risk of falls and fractures associated with vitamin D deficiency in older populations.^5^

Several vitamin D‐specific sun exposure guidelines/recommendations are currently available in Australia.^20^ However, Australia has the highest global prevalence of melanoma due to the large proportion of fair‐skinned populations residing in high UV exposure areas. Therefore, vitamin D supplementation (on its own or combined with calcium) is also recommended for a high‐risk population. However, evidence from randomized control trials (RCTs) that have considered vitamin D supplementation for falls and fracture prevention has been conflicting.^21^ Recent publication of a large vitamin D trial reported that vitamin D_3_ supplementation did not lower the risk of fracture compared to the placebo among generally healthy middle‐aged and older adults.^22^ While treatment for severe vitamin D deficiency remains essential, the necessity of vitamin D supplementation, especially to those already with adequate levels of vitamin D, is currently under debate. Several large trials have reported that supplementation of vitamin D‐replete populations did not provide significant beneficial effects on bone or extra‐skeletal health and falls risk.^21^ Our findings showed that engaging in multiple healthy lifestyle behaviours was associated with higher odds of having plasma 25OHD ≥75 nmol/L, suggesting a positive effect of healthy lifestyles on maintaining vitamin D status at relatively higher levels for older men.

A previous study in the same cohort found that plasma 25OHD <50 nmol/L was associated with increased risk of frailty and all‐cause mortality.^23^ However, evidence of a beneficial effect of maintaining circulating 25OHD levels higher than 75 nmol/L in this population is lacking, thus optimal vitamin D status remains uncertain and use of vitamin D supplementation in this population should be carefully assessed for benefit versus risk. Our current findings potentially strengthen the ‘reverse causality’ hypothesis popularized by Naveed Sattar, whereby higher 25OHD levels may be a consequence of, rather than a contributor to, generally better health.^24^

We found an association between high physical activity levels and lower odds of vitamin D insufficiency. This finding was consistent with previous epidemiological studies and with RCTs that showed regular exercise exerted a positive effect on vitamin D status in elderly population^25^, ^26^ which could be attributable in part to increased time spent outdoors during exercise. Preclinical studies support a role for muscle activity in vitamin D metabolism, with expression of the vitamin D receptor in skeletal muscle^27^ and 25OHD transit into skeletal muscle cells.^28^ These data support the notion that regular exercise may have a positive effect on maintaining vitamin D sufficiency, particularly in older populations at increased risk of sarcopenia and osteoporosis.

Surprisingly, we found no association between frequent fish consumption and vitamin D status in this population. This may be explained by the low overall fish consumption in this population, with only 11.3% men reporting fish intake at least three times per week. The low consumption of fish was consistent with the reports in the National Nutrition Survey in Australia conducted in 1995.^29^ Moreover, the vitamin D contents can vary by fish species and cooking methods,^30^, ^31^ and that information was not collected in our questionnaire. Given the positive relationship between oily fish consumption and improved vitamin D status^32^ and cardiovascular health outcomes^33^ in older populations, optimal fish consumption should be recommended.

Strengths of the present study are the large sample size of older men, with extensive baseline information to investigate the associations of interest in multivariable analyses. There are several limitations in our study. First, men included in the present analysis had participated in an earlier wave of the study, thus, a healthy survivor effect may be present. Men who were able to complete the follow‐up assessment had fewer self‐reported comorbidities and were more likely to have better health compared to those who did not reattend,^34^ which may be reflected in the relatively high 25OHD concentrations in this cohort. Second, this was an observational study with lifestyle questionnaires and blood samples collected at separate time‐points and no serial measurements. Therefore, we cannot draw conclusions regarding causality. Third, 25OHD blood levels could be affected by the amount of sun exposure, and that information was not included in the current analysis. It is worth noting that Australia is a country with abundant sunlight and these findings may not necessarily apply to populations in other geographical locations, particularly those at latitudes where there is less sunshine, and where physical activity is predominantly undertaken indoors. Also, misclassification due to differences between serum and plasma 25OHD concentrations should be considered. Furthermore, parathyroid hormone was not assayed in this population. The categorization of excessive alcohol consumption was based on the original lifestyle scoring, and lower thresholds may be more appropriate for future studies.^35^ Similarly, categorization of milk consumption prioritized avoidance of condensed or full cream milk and did not dissect out avoidance of milk consumption. Lastly, as only men aged 65 years and older were invited to the HIMS, findings from the present analysis may not apply to women and younger men.

In summary, in this population of healthy community dwelling older men, engaging in a range of healthy lifestyle behaviours including regular exercise, not smoking, maintaining normal body weight and healthy dietary habits, was associated with better vitamin D status. Although this population of men had a relatively high average plasma 25OHD, a fifth of the men had plasma 25OHD <50 nmol/L, indicating scope for interventions to optimize vitamin D status. Achieving sufficient physical activity may be an important strategy for improving vitamin D and musculoskeletal health in older men.

## AUTHOR CONTRIBUTIONS

**Xiaoying Liu**: Conceptualization, writing—original draft, formal analysis, writing—review and editing, visualization. **Kaye E. Brock**: Writing—original draft, writing—review and editing. **Tara C. Brennan‐Speranza**: Conceptualization, writing—original draft, writing—review and editing, supervision. **Leon Flicker**: Writing—review and editing, project administration. **Jonathan Golledge**: Writing—review and editing, project administration. **Graeme J. Hankey**: Writing—review and editing, project administration. **Christian M. Girgis**: Writing—original draft, writing—review and editing, supervision. **Bu B. Yeap**: Writing—original draft, writing—review and editing, supervision, project administration, data curation.

## CONFLICT OF INTEREST STATEMENT

The authors declare no conflict of interest.

## Data Availability

Due to privacy considerations the data sets analysed during the current study are not publicly available. Further information is available from the corresponding author upon reasonable request.
